# Implementation research on enhanced community case management of pneumonia in Bangladesh: study protocol

**DOI:** 10.7189/jogh.16.05001

**Published:** 2026-03-13

**Authors:** Shafiqul Ameen, Sadman Sowmik Sarkar, Mahin Bin Hamid, Md Ishtiak Anam Nobel, Sabit Saad Shafiq, Md Al-Mahmud, Azim Uddin AFM, Abid Anwar, Md Dudu Mia, Sharif Uddin Lotus, Anisuddin Ahmed, Hasan lbna Amin, Husam Md Shah Alam, Ashfia Saberin, Palash Kumar Saha, Sabina Ashrafee, Goutom Banik, Md Jahurul Islam, Shamim Ahmad Qazi, Shams El Arifeen, Ahmed Ehsanur Rahman, Yasir Bin Nisar

**Affiliations:** 1 International Centre for Diarrhoeal Disease Research, Bangladesh, Dhaka, Bangladesh; 2 Directorate General of Health Services, Ministry of Health and Family Welfare, Dhaka, Bangladesh; 3 Save the Children, Dhaka Bangladesh; 4 Indepenent Consultant, Geneva, Switzerland; 5 World Health Organization, Geneva, Switzerland

## Abstract

**Background:**

Previous trials in Africa and Asia, including Bangladesh, showed that community health workers can effectively treat young infants (7–59 days) with fast breathing and children (2–59 months) with chest indrawing pneumonia at home with oral amoxicillin using enhanced integrated community case management (iCCM) protocols. However, the Enhanced Management of Pneumonia in Community (EMPIC), its pneumonia-specific component, has not yet been applied in routine government health systems. Here, we developed a protocol for a feasibility study on the integration of EMPIC into community clinics in Bangladesh through existing government health systems.

**Methods:**

This study will adopt an implementation research approach that integrates quantitative and qualitative methods. A delivery package will be co-developed with stakeholders to introduce enhanced pneumonia management through government systems in three phases within community clinics of selected *upazilas* in Kushtia and Dinajpur districts. We will use the plan-do-check-act cycle framework to evaluate implementation, track progress, identify gaps, and test potential solutions. Data collection methods will include health facility assessments, data extractions from routine registers and monthly reports, household surveys, and community follow-ups of under-five children with pneumonia on days 7 and 14 post-treatment. The primary outcome is high (*i.e.* ≥80%) and effective coverage (*i.e.* patients receiving the full course of pneumonia treatment) of pneumonia treatment in under-five children. Secondary outcomes include treatment failure rates among under-five children with pneumonia; availability of commodities and supplies for pneumonia management; health worker knowledge, caregiver awareness and care-seeking practices regarding childhood pneumonia; pneumonia prevalence among under-five children; community clinic utilisation for pneumonia-related symptoms by under-five children; and treatment compliance.

**Conclusions:**

Our findings may inform the evidence-based scale-up of enhanced pneumonia management in Bangladesh and other low- and middle-income countries, contributing to improved community-level management of childhood pneumonia.

Despite progress in reducing child mortality over the past two decades, global efforts fell short of achieving the Millennium Development Goal 4 target of reducing under-five mortality by two-thirds from 1990 to 2015 [1]. Despite further drops in the global under-five mortality rate by 2020 [1], an estimated 4.8 million were recorded among this population in 2023, translating to 13 100 deaths a day [2].

Pneumonia accounts for approximately 14% of deaths among children under five years of age, making it the leading cause of death in this group [3]. In 2019, there were 45 million cases of pneumonia globally among children under five years old, resulting in over 700 000 deaths. However, the burden and mortality of childhood pneumonia are unevenly distributed worldwide: sub-Saharan Africa, for example, has twice the incidence and 60 times the mortality of high-income countries [4]. Accelerated efforts are needed to avert pneumonia-related deaths to achieve the highly ambitious Sustainable Development Goal (SDG) target of reducing under-five mortality to below 25 per thousand live births [5].

In Bangladesh, pneumonia accounted for around 24% of all under-five deaths in 2022, with a mortality rate of 7 per 1000 live births [6]. Despite a high coverage of *Haemophilus influenzae* type b and pneumococcal conjugate vaccines (98–99%), only 46% of children with suspected pneumonia are taken to an appropriate healthcare provider [7]. In 2022, the country’s median integrated Global Action Plan for Pneumonia and Diarrhoea score was 68% overall and 81% for pneumonia specifically, falling short of the global target of 84% [7]. The care-seeking behaviour of its population is influenced by factors such as caregivers' knowledge, parental joint decision-making, mistrust of the healthcare system, faith in traditional care, spiritual beliefs, distance to health facilities, shortage of qualified staff, and household wealth [8–10]. Many children with pneumonia symptoms are taken to private sector providers, with only a small percentage seeking care from public health facilities [6]. The gaps in achieving high coverage of core interventions and the reliance on inappropriate care-seeking practices highlight the need for improved community-level pneumonia management in Bangladesh.

Global recommendations for low-resource settings indicate the adoption of the Integrated Management of Childhood Illness (IMCI) strategy for outpatient management of common childhood illnesses, including pneumonia. Developed by the World Health Organization (WHO) and the United Nations Children’s Fund (UNICEF) in the mid-1990s, the IMCI strategy promotes accurate identification of childhood illnesses in outpatient settings, appropriate combined treatment of all major illnesses, strengthened counselling of caretakers, and provision of preventive services [11–13]. It also emphasises the need for speeding up the referral of severely ill children and improving the quality of care at the referral level [11,13–15]. In home settings, it promotes appropriate care-seeking behaviours, improved nutrition, preventive care, and correct implementation of prescribed care [11,13–15].

Effective implementation of IMCI has led to increased use of health services, improved health workers' performance and quality of care, reduced irrational use of drugs, and a decline in under-five mortality rates in countries with limited resources [16–24]. The approach is now implemented in more than 100 countries, with 81% reporting full implementation of all three components, and approximately half reporting adoption in 90% or more districts [15,25]. Despite the high reported implementation, IMCI has yet to benefit children most in need, as coverage remains lowest in high-mortality settings, as do gaps in availability, accessibility, and quality of services for pneumonia management [7,15,26]. Achieving the 2025 target of ending preventable pneumonia deaths and the 2030 SDG target of reducing neonatal mortality to 12 or fewer per 1000 live births will be challenging without revitalised strategic focus, substantial investments, and concerted efforts across the global health community [5,7].

Bangladesh was one of the five countries targeted by the WHO-led Multi-Country Evaluation of IMCI Effectiveness, Cost, and Impact study [27]. In its last two years, the mortality rate was 13.4% lower in IMCI intervention areas compared to comparison areas [17]. The study also reported that successful implementation of the IMCI package led to improved health worker skills, health system support, and family and community practices, translating into increased care-seeking for illnesses [17]. It also demonstrated a positive effect on exclusive breastfeeding and stunting [17]. Based on the experience gained and evidence generated through this study and global recommendations, the Government of Bangladesh adopted the IMCI strategy in 1998. After successful piloting of facility-based IMCI in three sub-districts (*upazillas*), IMCI was included in the Sector Investment Plan of the Health, Nutrition, and Population Sector Programme 2005–2010 as one of the priority strategic components to improve child health in Bangladesh. Currently, facility-based IMCI has been scaled up across the country.

Addressing the high burden of childhood pneumonia in Bangladesh, however, requires further expanding the coverage of IMCI services and hospital inpatient care. The current service delivery model, starting at union-level facilities (health centres), is inadequate, with less than 50% coverage [7]. To improve this, services need to be extended to community clinics (CCs) at the ward/village level, each serving 6000–10 000 people. As of June 2020, over 13 812 CCs have been established and made functional, staffed by community healthcare providers (CHCPs) with basic training [28].

Evidence from multi-country trials in Ethiopia, Malawi, Bangladesh, and India shows that community-based management of pneumonia with oral amoxicillin is as effective as hospital referrals, with treatment failure rates being equivalent and deaths observed in less than 1% of cases [29,30]. Effective pneumonia treatment requires early case identification, prompt treatment close to home, well-trained providers, necessary supplies, and efficient referral systems. High non-compliance with referral advice in LMICs underscores the need for community-based management [7,20,21,31–33].

Implementing revised pneumonia management guidelines [34] in CCs can reduce the need for referrals, decrease costs and save time. Assessing the feasibility of this approach in real-life settings is crucial before scaling up. This will help improve pneumonia treatment in areas where access to referral facilities is limited, enhancing coverage and effectiveness and ultimately reducing childhood pneumonia burden. Therefore, through an implementation research approach, we aim to introduce Enhanced Management of Pneumonia in Community Clinics (EMPIC) through the government health systems and structures in Bangladesh to increase effective population-based coverage of pneumonia treatment in under-five children and scale-up.

## METHODS

The goal of this study is to achieve high and effective population coverage of pneumonia treatment in under-five children in Bangladesh and to develop a scalable implementation strategy for contextual adaptation to other low- and middle-income countries (LMICs).

Our primary objective is to increase access to pneumonia treatment in under-five children to achieve high (≥80%) and effective population-based coverage (*i.e.*, patients receiving the full course of pneumonia treatment) in the study area. We also set five secondary objectives:

1. to increase care-seeking practices from appropriate healthcare providers for general danger signs and pneumonia-related signs of under-five children;
2. to determine the rate of clinical treatment failure of young infants 7–59 days old with fast breathing and children 2–59 months old with fast breathing and/or chest indrawing pneumonia who received treatment from CHCPs;
3. to assess the compliance with pneumonia treatment among young infants 7–59 days old with fast breathing and children 2–59 months old with fast breathing and/or chest indrawing pneumonia who received treatment from CHCPs;
4. to explore the knowledge, perceptions, and experiences of health facility staff, CHCPs, and beneficiaries on the implementation of pneumonia management;
5. to improve the availability of essential commodities in the CCs to manage pneumonia in under-five children.

We will achieve these objectives through three activities: designing an implementation delivery package for introducing the EMPIC in Bangladesh (activity 1); employing a continuous learning process during the implementation period to understand the prerequisites for the scale-up of the package (activity 2); and assessing whether the implementation delivery package worked by documenting the implementation process, understanding and assessing care practices, and exploring the experiences of health managers, health service providers, and caregivers related to the implementation of the package (activity 3).

### Study design

This study will use an implementation research approach, incorporating both quantitative and qualitative data collection methods. The design will be informed by evidence from a cluster-randomised non-inferiority trial conducted in several countries, including Bangladesh, which demonstrated that community-based treatment of young infants (7–59 days) with fast-breathing pneumonia by trained community health workers is both safe and effective, performing comparably to referral-based management [29,30]. Building upon these findings, this study focuses on evaluating the implementation outcomes in real-world health system contexts by integrating EMPIC within routine primary healthcare delivery systems.

### Study settings, period, and population

We will select two *upazilas* (sub-districts) in total: one each from the Kushtia and Dinajpur districts. From them, we will choose 20 CCs based on service availability and caseload, in consultation with national and district-level health managers. Dinajpur district covers an area of 3437.98 km^2^ and is home to 299 0128 inhabitants, resulting in a population density of 868 people per km^2^ [35]. The district has one medical college hospital, one district hospital, 12 *upazila* health complexes (sub-district hospitals), 119 union sub-centres and union health & family welfare centres (union level health centres), and 337 CCs (health outpost centres) [36]. Kushtia district has a population of 1 986 838 and covers an area of 1621.15 km^2^, leaving a density of 1073 people per km^2^ [37]. The district's health facilities include one district hospital (secondary level referral hospital), five *upazila* health complexes (sub-district hospitals), 73 union health and family welfare centres (union level health centres), and 205 CCs [36]. The study will be conducted across 24 months, where 18 months will be reserved for the delivery of the implementation package.

The study population will be under-five children presenting with pneumonia in CCs, CHCPs working in CCs, caregivers and family members of children visiting CCs, district and sub-district level health managers and supervisors and central-level health managers and policymakers.

### Data collection by activities

#### Activity 1

**Figure 1:**
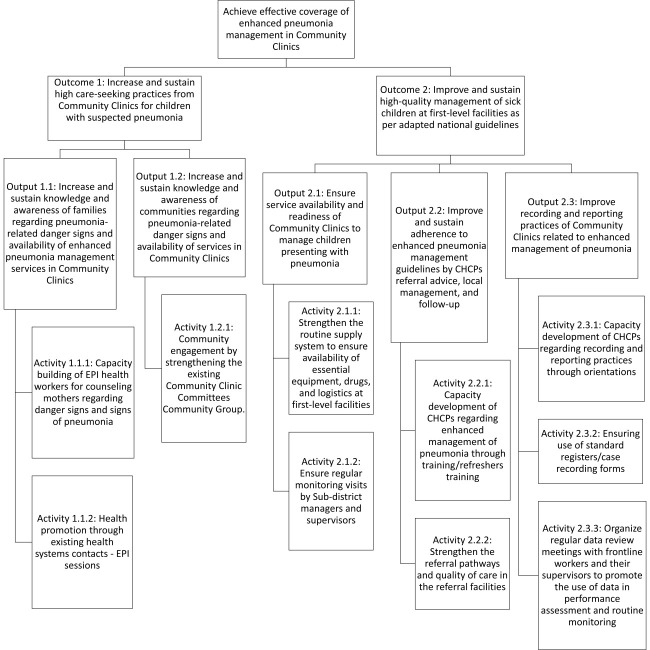
Implementation plan for pneumonia management in community clinics. CHCP – community healthcare provider, EPI – Expanded Programme of Immunization.

We propose to develop an implementation package for introducing EMPIC through consultation with key stakeholders in Bangladesh, including the National Newborn Health and IMCI Programme, the Community-based Healthcare Programme, and other development partners. The implementation package will be developed around the enhanced management of pneumonia strategy (**Figure 1**), where the integrated community case management (iCCM) strategy [34], recommended by the WHO for community health workers, will be modified and incorporated into the existing IMCI strategy [38] framework in Bangladesh. Under the enhanced strategy, under-five children presenting with any danger signs (severe pneumonia or very severe disease/critical illness), infants aged 0–6 days with fast breathing or possible serious bacterial infection, and infants aged 7–59 days with any danger sign (possible serious bacterial infection) will be directed to referral facilities. In addition, CHCPs will conduct pulse oximetry in children with cough and/or difficulty in breathing and refer hypoxaemia cases to referral facilities. In contrast, children aged 7–59 days with fast breathing pneumonia or aged 2–59 months with fast breathing and/or chest indrawing pneumonia will be treated with oral amoxicillin (**Box 1**).

Box 1 **Signs and classification of sick young infants and sick children, by different age groups, according to the IMCI Chart Booklet (Bangladesh) 2019 [38]**

0–59 days

Possible serious bacterial infection or very severe disease- critical illness

*Any one or more of the following signs: unconsciousness/drowsy, convulsion or H/O convulsion, unable to feed, persistent vomiting, bulging fontanelles, apnoea, central cyanosis, major bleeding, weight <1500 g, major congenital malformation, surgical condition requiring hospitalisation*

Possible serious bacterial infection or very severe disease – clinically severe infection

*Any one or more of the following signs: severe chest indrawing, fever (37.5°C or above) or low body temperature (less than 35.5°C), not feeding well,* *movement only when stimulated/no movement at all*

Possible serious bacterial infection or very severe disease – fast breathing pneumonia (0–6 days)

*Fast breathing (60 breaths per minute or more) for age 0–6 days.*

*Fast breathing pneumonia (7–59 days).*

*Fast breathing (60 breaths per minute or more) for age 7–59 days.*

**2–59 months**

Severe pneumonia or very severe disease

Any general danger signs: the child being unable to drink or be breastfed, vomiting everything, having convulsions, being lethargic or unconscious, and convulsing now, or stridor in a calm child, or oxygen saturation (SpO_2_) <90%

Pneumonia

Chest indrawing or fast breathing (50 breaths per minute or more for age 2–12 months, 40 breaths per minute or more for age 12–59 months)

To ensure the safe and effective use of pulse oximetry within CCs, devices will undergo routine maintenance and calibration, and supplies of drugs and equipment will be coordinated through the Directorate General of Health Services logistics system. The CHCPs will receive periodic refresher training to maintain competency in pneumonia management, with an emphasis on rational antibiotic use and strict adherence to referral protocols. Antibiotic prescriptions and referral decisions will be regularly audited and monitored through routine follow-up visits, while plan-do-check-act (PDCA) cycles will enable identification and correction of any deviations in real time, collectively reducing the likelihood of over-treatment, inappropriate antibiotic use, or missed referrals. These operational safeguards, combined with the PDCA cycles for continuous learning and ongoing stakeholder engagement, will allow the programme to monitor and address potential challenges related to device function, supply reliability, and rational antibiotic use. Insights from the implementation activities will inform ongoing improvements to the programme model through the PDCA cycles in activity 2, ensuring continuous adaptation and context-specific improvements.

The intervention package will include activities aimed at increasing care-seeking practices for childhood illnesses at CCs. The first set of interventions will focus on raising knowledge and awareness among families and communities about the danger signs. The second set will aim to strengthen health systems and improve the quality of care for children with pneumonia at CCs. While these health system interventions are not directly linked to increasing demand, ensuring the availability, readiness, and quality of services is essential for enhancing care-seeking practices.

#### Activity 2

**Figure 2:**
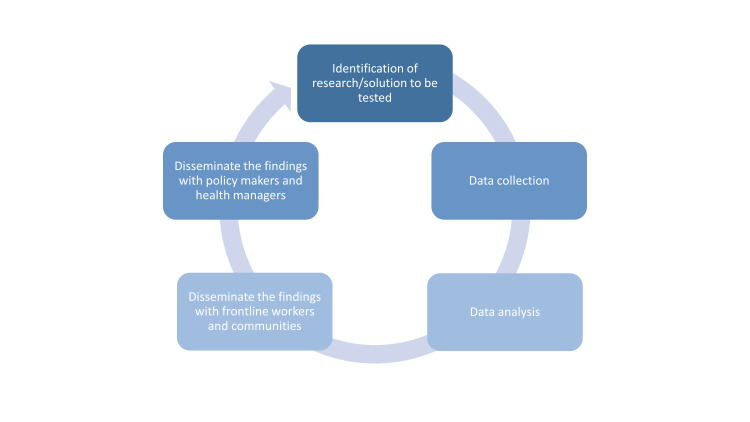
Proposed plan-do-check-act cycle.

The logic model for enhanced pneumonia management in CCs will be finalised through consultations with key stakeholders, including national policymakers, clinical experts, and health managers. The IMCI programme will lead the implementation, using government systems without financial incentives for providers. The implementation research team (icddr,b) will support the IMCI programme and continuously update the package. An initial situation analysis in Kushtia and Dinajpur districts will inform updates to the programme model. The feasibility will be tested in three rounds using the PDCA cycle (**Figure 2**). Implementation findings will be reviewed in existing monthly sub-district health facility meetings with CHCPs in the study area, which will be chaired by an *upazila* health and family planning officer. These sessions, embedded in a formal governance platform, will track progress, identify gaps, and agree on corrective actions, ensuring accountability, while avoiding parallel structures. To monitor and mitigate potential increases in CHCP workloads, task feasibility workflow challenges, and adherence to the enhanced pneumonia protocol will be assessed through PDCA cycles and routine supervision. Feedback from CHCPs will inform minor adjustments or supportive measures to maintain protocol fidelity without compromising service delivery or sustainability. Stakeholders will be involved at every stage to ensure ownership and capacity development. These minor adjustments may be introduced in real time, while any substantive protocol changes will require prior institutional review board approval. Both qualitative and quantitative data will be collected by a dedicated research team.

**Figure 3:**
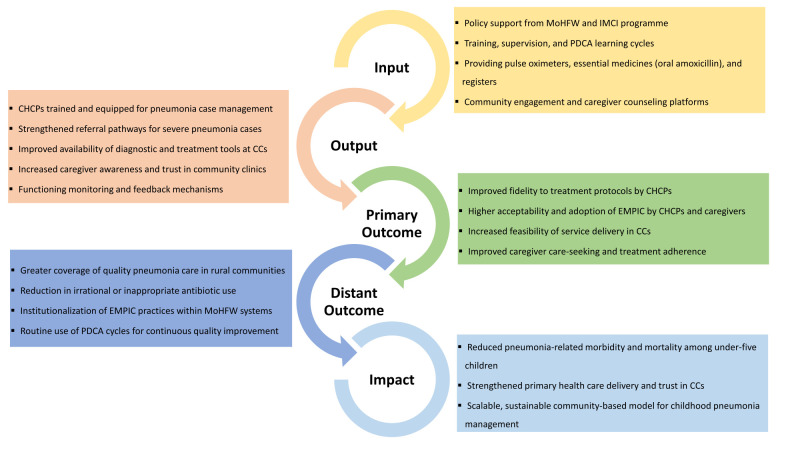
Theory of Change underpinning EMPIC intervention. CC - community clinic, CHCP – community healthcare provider, EMPIC - Enhanced Management of Pneumonia in Community, IMCI – Integrated Management of Childhood Illness, MoHFW – Ministry of Health and Family Welfare, PDCA – Plan-Do-Check-Act.

The EMPIC intervention is guided by a theory of change (**Figure 3**) that links CC-level pneumonia management to improved health outcomes. The framework outlines how key inputs, such as training, supervision, diagnostic tools, and community engagement, are expected to strengthen service delivery outputs, leading to improved treatment fidelity, care-seeking, and adoption of enhanced pneumonia management. These primary outcomes are anticipated to translate into greater coverage, integration within health systems, and, ultimately, reduced pneumonia-related morbidity and mortality among under-five children.

#### Activity 3

Health facility assessments will be conducted at baseline and end-line to evaluate the service availability and readiness of CCs, using structured tools to assess equipment, drugs, and logistics for pneumonia management, as well as CHCPs’ knowledge regarding management of childhood pneumonia. We will extract data from routine registers to evaluate recording and reporting practices, and will monitor monthly utilisation reports through central management information systems. Community follow-up of the pneumonia-classified children on the 7th and 14th days of CC visit will involve household visits by study-nurses to assess treatment failure rates and adherence to guidelines. Baseline and endline household surveys will measure caregivers' knowledge, care-seeking practices, and treatment outcomes, defining treatment failure based on specific clinical criteria.

We will explore the experiences of health managers, CHCPs, and caregivers through in-depth and key informant interviews which will focus on contextual factors, including social determinants of health, cultural beliefs, caregiver practices, and CHCPs and health system constraints (such as workload assessment) that influence care-seeking and treatment adherence. Insights from these interviews will inform iterative refinement of the intervention and interpretation of fidelity, acceptability, and adoption outcomes. This comprehensive data collection (**Table 1**) will inform continuous improvements in pneumonia management at CCs.

**Table 1:** Data collection procedure throughout the study period

	Baseline	Q1	Q2	Q3	Q4	Q5	Q6	Endline
Health facility assessment	X	O	O	O	X	O	O	X
Knowledge assessment of service providers	X	O	O	O	O	O	O	X
Household survey	X	O	O	O	O	O	O	X
Data extraction	O	X	X	X	X	X	X	O
Community follow-up	O	X	X	X	X	X	X	O
Qualitative exploration	X	X	X	X	X	X	X	X

X – active data collection period for the specified activity, O – no data collection (activity not conducted during that time period

To ensure systematic monitoring of the implementation process, we will align the EMPIC activities with the WHO implementation research outcome variables (**Table 2**). This approach allows us to evaluate not only the outcomes of the intervention, but also how it is delivered and adopted within routine health system settings.

**Table 2:** EMPIC process indicators and data collection by WHO implementation outcome

IR outcome	Definition	Measurement and data Source
Fidelity	Adherence to protocol for pneumonia classification and treatment, and referral for danger sign cases	Review of routine registers
Acceptability	Whether the enhanced management of pneumonia is accepted by caregivers, service providers, and health managers	Qualitative interviews and stakeholder consultations
Adoption	Initial uptake of enhanced pneumonia management by CHCPs	Qualitative interviews and routine register reviews
Coverage	Proportion of eligible children receiving enhanced management of pneumonia by month/quarter	Review of routine registers

CHCP – community healthcare provider

### Study outcome

The primary outcome of interest is high (≥80%) and effective (patients receiving the full course of pneumonia treatment) coverage among under-five children (**Table 3**). The secondary outcome of interests are: treatment failure rate among young infants 7–59 days old with fast breathing and children 2–59 months old with chest indrawing and/or fast breathing pneumonia who received treatment from CCs; availability of essential commodities and supplies for managing childhood pneumonia; CHCPs’ knowledge of managing childhood pneumonia; caregiver’s knowledge and care-seeking practices regarding childhood pneumonia; and prevalence of pneumonia among under-five children, utilisation of CCs for pneumonia related symptoms; and treatment compliance.

**Table 3:** Study enrolment and outcome assessment strategy

Activity	What	Who	When	Where	How
Case identification	Identify young infants with fast breathing pneumonia and children aged 2–59 months with fast breathing and/or chest indrawing pneumonia from data extracted from the service registers	Trained data extractors appointed by the study team	Twice in a week	Health facilities (CCs and union-level health facilities)	Trained data collectors will extract data from the registers or case record forms in the health facilities. From there, they will identify young infants with fast breathing pneumonia and children aged 2–59 months with fast breathing and/or chest indrawing pneumonia. They will provide this information to study-appointed nurses/paramedics who will conduct enrolment of the participants.
Screening and Enrolment	Enrol eligible young infants and children aged 2–59 months based on inclusion and exclusion criteria	Study appointed nurses or paramedics	One to three days after facility visit	Phone	Nurses and paramedics will screen the children based on the inclusion and exclusion criteria from the information received from study-appointed data extractors. They will contact the caregivers of the eligible children over the phone and take verbal consent for the home visit. They will enrol the children if consent is given by the caregivers.
Treatment provision and treatment documentation	A tool to document treatment provided to young infants with fast breathing pneumonia and children aged 2–59 months with fast breathing and/or chest indrawing pneumonia	Trained data extractors appointed by the study team	Twice in a week	Health facilities (CCs and union-level health facilities)	Trained data collectors will extract data on treatment provided by the health workers from the registers or case record forms in the health facilities
Treatment follow-up and outcome assessment	A tool to assess adherence to treatment given to enrolled young infants and children aged 2–59 months	Study-appointed nurses or paramedics	Day 7 and 14 of enrolment (visiting health facilities- CCs and union-level facilities)	Home	Trained nurses/paramedics will conduct home visits on days 7 and 14 of enrolment. They will interview caregivers to explore adherence to the treatment given to enrolled young infants and children aged 2–59 months. They will also assess the treatment given to enrolled young infants and children aged 2–59 months to explore treatment failure rate.

CC – community clinics

Treatment failure will be defined as: death anytime within 14 days of initial assessment; persistence of the fast breathing in young infants 7–59 days or persistence of chest indrawing and/or fast breathing in children 2–59 months old on day 7 of enrolment; any indication of hospitalisation (that is, with any sign of clinical deterioration) on day 7; and development of serious adverse event of the treatment (anaphylactic reaction, severe diarrhoea, disseminated and severe rash).

### Sample size and sampling strategy

#### Household survey

Baseline data from the Bangladesh Health and Demographic Survey 2017–18 indicate a 40% care-seeking rate among children under five with suspected pneumonia in the four weeks preceding the survey [39]. Assuming a baseline value of care-seeking practice for childhood illness among children under five years of age with suspected pneumonia in the past four weeks is 40% in baseline and (based on our objectives) 80% in end-line, and setting a 5% margin of error and 95% confidence interval, the unadjusted sample size is 23 children under five years of age with suspected pneumonia per district, increasing to 58 after adjustment for a 20% non-response rate and a design effect of 2.0. To achieve this sample size, we will need to reach 5121 households and 20 480 population per district. For the household survey, we will use a stratified sampling technique to ensure equal representation. Specifically, we will select villages, considered as the primary sampling unit, through probability proportional to size sampling, after which we will identify and interview all women with under-five children in the sampled villages.

#### Community follow-up

In the community follow-up assessing treatment failure rates, the EMPIC cluster randomised controlled trial (which included Bangladesh) reported a 5.4% treatment failure rate among children aged 7–59 days with fast breathing pneumonia [29] and a 4.3% failure rate among children aged 2–59 months with chest indrawing pneumonia [30]. Here, we will assume slightly higher treatment failure rates in real-life settings: 8% for children aged 7–59 days with fast breathing pneumonia, and 6% for children aged 2–59 months with chest indrawing pneumonia.

Using the standard formula for a single proportion, a 4% margin of error, and 95% confidence level, we arrive at a required sample size of 176 non-hypoxaemic young infants aged 7–59 days with fast breathing pneumonia for descriptive analysis per site. Assuming 5% loss to follow up, we will enrol a total of 185 non-hypoxaemic young infants with fast breathing aged 7–59 days, given oral amoxicillin within our study and followed up for outcome assessment at each district.

Lastly, using the standard formula for a single proportion with a 3% margin of error and a 95% confidence level, we calculated a required sample size of 238 non-hypoxaemic children aged 2–59 months with chest indrawing pneumonia per site. Assuming 5% loss to follow-up, we will enrol 250 non-hypoxaemic 2–59 months old children with chest indrawing pneumonia, given oral amoxicillin in our study and followed up for outcome assessment at each district.

#### Qualitative explorations

We will determine the sample size for the qualitative segment of the study through data saturation. As a general guide, we propose conducting 8–10 interviews per round with frontline health workers, 10–15 with family members, 3–4 with district-level managers and supervisors, and 3–6 with central-level managers and policymakers.

#### Other data collection methods

We will extract data from service registers twice a week in all CCs throughout the study implementation period. We will also assess all CCs from the study sites and interview all CHCPs for knowledge assessment at baseline and endline.

### Quality control and assurance

We will ensure data quality through regular monitoring and data checks. Two observers will independently validate 2% of all records, with continuous feedback throughout the data collection process to the data collectors. For quality assurance, staff from the icddr,b office, along with designated field managers and personnel, will closely monitor the data collection process, while the WHO team will conduct on-site visits to oversee and support field-level implementation.

### Data security and ownership

We will assign individual identifiers to link study records or use an algorithm based on names and birth dates, where identifying information will be removed post-matching. These anonymised data will be uploaded weekly to a central web-based platform coordinated by icddr,b, and will be stored on a secure server with automatic nightly backups to an off-site location. The data will be encrypted, accessible only to the research team members. The data generated will be jointly owned by icddr,b and the WHO, and any use will require consent from both parties. In accordance with icddr,b’s data access and archival policies, they will become available for future research three years after the study completion, as recorded by the institutional review board of icddr,b, unless WHO or icddr,b specifies a shorter release period.

### Data analysis

We will use descriptive statistics to report the readiness of CCs for pneumonia case management, knowledge of CHCPs, utilisation of CCs by under-five children, and treatment compliance by caregivers, treatment failure rates, knowledge of caregivers regarding general danger signs and pneumonia danger signs, and care-seeking by caregivers for under-five children. Changes in these indicators will be presented quarterly. We will score health facility readiness based on the availability of essential items for managing pneumonia cases according to national guidelines and consultations with the clinical experts and national health managers. We will categorise facility readiness based on item availability as poor (0–50%), moderate (50–75%), and good (75–100%).

We will calculate treatment compliance based on caregiver recall of giving prescribed antibiotics from CCs at home, collected through the follow-up interviews with caregivers. Data will be analysed using Stata, version 16.0 (StataCorp LLC, College Station, Texas, USA).

We will base our qualitative analysis on a thematic approach. First, multiple researchers will code a minimal set of transcripts to establish intercoder reliability, after which a master code-list will be created for the remaining transcripts. We will compare the new and earlier data iteratively data to identify similarities and differences.

### Dissemination plan

Upon completion, we will publish the study results in a peer-reviewed journal and present them at relevant national and international conferences and workshops. This will facilitate knowledge sharing and discussions with healthcare professionals, policymakers, and researchers, as well as provide evidence-based insights for pneumonia management in CCs. We will also develop a report on the study's methodology, findings, and recommendations for sharing with stakeholders such as government agencies, healthcare providers, and non-governmental organisations involved in child health.

## DISCUSSION

This implementation research aims to assess the feasibility of introducing EMPIC within CCs through the existing governmental health systems in Bangladesh. The goal is to improve population-based coverage of pneumonia treatment among children under the age of five and facilitate scale-up. Pneumonia-related deaths predominantly occur at the community level, where the WHO and UNICEF have established the iCCM approach for community health workers. According to the previous iCCM protocol, children aged 2–59 months presenting with fast breathing alone should receive oral amoxicillin, while those exhibiting chest indrawing or other danger signs should be referred to higher-level health facilities. Similarly, young infants aged 0–59 days with fast breathing alone or any danger sign should be directed to referral facilities by community health workers [40]. Subsequently, the WHO conducted a cluster-randomised, community-based trial across four countries in Asia and Africa, including Bangladesh, to evaluate whether community health workers could manage young infants aged 7–59 days with fast breathing alone, or children aged 2–59 months with chest indrawing pneumonia, using oral amoxicillin at home without referrals (enhanced iCCM strategy). The trial demonstrated that treating non-hypoxaemic, non-severe pneumonia with oral amoxicillin administered by community health workers was non-inferior to the standard iCCM referral-based approach [29,30]. Bangladesh is currently implementing the globally endorsed IMCI strategy, developed by the WHO and UNICEF, to manage common childhood illnesses, including pneumonia, in outpatient settings [39]. This study aims to integrate the enhanced iCCM approach into the existing IMCI framework operational in Bangladesh.

The early implementation of this strategy within a real-world programmatic context is crucial for enabling parents, community health workers, and policymakers to recognise the advantages of the enhanced iCCM approach in managing childhood pneumonia. This initiative aims to improve access to pneumonia treatment closer to home, with the anticipated benefits being: minimising the need for referrals or hospital admissions; reducing costs associated with treatment and its administration; lowering family expenses related to transportation, food, and wage loss; saving time for both families and health workers; and alleviating the burden on already overwhelmed hospital facilities.

One of the key interventions in this study is the detection of hypoxaemia by community health workers using pulse oximetry. Bangladesh is among the first LMICs to incorporate pulse oximetry into outpatient pneumonia management, following global recommendations [41,42]. However, this approach has not yet been implemented at community-level health facilities, such as CCs. Here, we will introduce pulse oximetry devices in CCs within the study areas and train community health workers on their appropriate use.

Bangladesh is among the few LMICs where community health workers have the provision to administer certain antibiotics, including oral amoxicillin. Nevertheless, consistent with global concerns, there are apprehensions in Bangladesh regarding the empirical use of antibiotics for pneumonia treatment, which may contribute to antibiotic resistance and inappropriate use in cases of viral infections [43,44]. Evidence indicates that the WHO's standard case management guidelines for pneumonia support the rational use of antibiotics in infants and children [45,46]. To mitigate the risk of improper use of oral amoxicillin by community health workers, their activities will be supervised and closely monitored by clinical supervisors.

Delayed or inadequate care-seeking remains a critical factor contributing to treatment failure and pneumonia-related mortality, particularly in resource-limited settings [47,48]. In Bangladesh, care-seeking behaviour from appropriate healthcare providers and facilities has consistently been suboptimal over the years [6]. Consequently, a key focus of the implementation package used in our study will be to improve and sustain care-seeking practices for pneumonia-related symptoms at the community level.

### Foreseeable strengths and limitations

A key strength of this study will be its implementation under the guidance of the Ministry of Health in Bangladesh. Policymakers at the national level, along with health managers from district and sub-district levels, community health workers, and their clinical supervisors, will actively participate in the planning and execution phases. Evidence from prior implementation research in Bangladesh has shown that sustained involvement of these stakeholders fosters a sense of ownership and facilitates skill development, which has proven instrumental in successfully integrating interventions into national policies and strategies [41,42,49].

This study might also have several limitations. First, the clinical diagnosis of pneumonia will not be confirmed through radiological or microbiological assessments due to the impracticality of conducting these evaluations in community settings. Second, fast breathing in young infants could be attributed to various conditions such as viral respiratory infections, high fever, bronchiolitis, or malaria, which are unlikely to respond to amoxicillin. At present, differentiating between viral, bacterial, or mixed causes of pneumonia at the time of initial assessment remains a challenge, particularly in community-based settings. Third, EMPIC’s participatory design, involving policymakers and health workers, may introduce selection bias or conflicts of interest. These risks will be mitigated through independent data collection, triangulation across multiple sources, standardised tools, and PDCA cycles, and the engagement of stakeholders, which will be essential for ownership and sustainability. Furthermore, the 18-month study period will limit the assessment of long-term changes in health-seeking behaviours, treatment coverage, and outcomes. However, this implementation research focuses on feasibility, fidelity, acceptability, and integration of EMPIC into routine health services, providing foundational insights for longer-term evaluation and scale-up. Moreover, while we aim to provide insights for adaptation in other low-resource settings by documenting key factors, such as workforce capacity, supply chains, and community acceptability, our findings will be context-specific to the selected *upazilas* in Bangladesh. Another limitation will be caregiver recall of antibiotic administration, which is subject to recall and social desirability bias. Caregivers will be asked to show the prescription and medicine bottles during follow-up visits on days 7 and 14 to confirm whether antibiotics were indeed prescribed to the children; however, this procedure will not be employed for assessing treatment compliance. As the follow-up visits will be conducted on days 7 and 14, we anticipate that it will minimise the recall bias, as the recall period is short. Finally, the evaluation framework will rely on routine register data, caregiver recall, and household surveys, which are vulnerable to reporting bias, recall bias, and data quality issues. To minimise these issues, we will triangulate information from multiple sources. Specifically, the CHCP registers will record detailed clinical and treatment data for all under-five children presenting to CCs. Study nurses will conduct structured household community follow-ups on days 7 and 14 to assess treatment adherence, antibiotic use, and clinical outcomes of the pneumonia children, cross-checking caregiver reports with register entries. Baseline and endline household surveys will capture caregiver knowledge, care-seeking practices, and treatment outcomes, while health facility assessments will monitor service readiness, equipment, and drug availability. Qualitative interviews with caregivers, CHCPs, and health managers will document implementation processes, challenges, and contextual factors. Standardised tools, rigorous data collector training, periodic audits, and PDCA cycles with twice-weekly site visits will promptly identify and resolve operational or data-quality issues. Together, these complementary monitoring and data collection strategies will provide high-quality data to guide EMPIC implementation and policy decisions, forming a structured process evaluation framework aligned with WHO implementation research outcomes, including fidelity, adoption, acceptability, appropriateness and coverage.

## CONCLUSION

The insights obtained from this implementation research can contribute to evidence-based strategies for improving the management of pneumonia in community settings within Bangladesh and other LMICs. These efforts are aligned with the goal of eliminating preventable childhood pneumonia-related deaths by 2030 and targets outlined in SDG 3.
